# From Evaluation to Elevation: Standardized Letter of Evaluation Domains Tied to Future Emergency Medicine Chief Residents

**DOI:** 10.5811/westjem.48713

**Published:** 2026-01-21

**Authors:** Abagayle Bierowski, Zaid Tayyem, Casey Morrone, Carlos Rodriguez, Chaiya Laoteppitaks, Peter Tomaselli, Dimitrios Papanagnou, Xiao Chi Zhang

**Affiliations:** * Thomas Jefferson University, Department of Emergency Medicine, Philadelphia, Pennsylvania; † Sidney Kimmel Medical College, Philadelphia, Pennsylvania; ‡ Thomas Jefferson University, Abington Hospital, Department of Emergency Medicine, Philadelphia, Pennsylvania; § Wake Forest University, Department of Emergency Medicine, Winston-Salem, North Carolina; || Wake Forest University School of Medicine, Winston-Salem, North Carolina; # Temple University, Chestnut Hill Hospital, Department of Emergency Medicine, Philadelphia, Pennsylvania

## Abstract

**Introduction:**

The Standardized Letter of Evaluation (SLOE) is a core component of emergency medicine (EM) residency applications, designed to assess clinical performance, professionalism, and leadership potential. While its utility in selecting residency candidates is well established, its association with future leadership roles, such as chief resident, remains unclear. Identifying early indicators of leadership potential could inform both recruitment and resident development efforts. In this study we aimed to evaluate whether medical students’ SLOEs are associated with subsequent selection as chief residents, offering insight into the SLOE’s potential to forecast future leadership within EM.

**Methods:**

We conducted a retrospective review of 243 de-identified SLOEs from 101 residents at a single urban, academic EM residency program between 2015–2021; 21 residents (20.8%) went on to hold chief resident roles between 2018–2024. The SLOEs were numerically scored across 10 groups. We excluded SLOEs lacking quantitative ratings or written for non-core EM rotations.

**Results:**

Chief residents scored significantly higher than non-chief residents in three of 10 evaluated domains following Bonferroni correction for multiple comparisons: teamwork (P = .002), overall comparison to EM applicants from prior years (P = .003), and anticipated rank-list placement (P = .004). No significant differences were found in domains such as clinical reasoning, communication skills, or commitment to EM. Sex distribution among chief residents was approximately equal, minimizing concerns for confounding.

**Conclusion:**

The Standardized Letter of Evaluation may offer limited but meaningful insight into future leadership potential in EM. Traits such as teamwork, self-directed learning, and perceived autonomy may distinguish future chief residents even prior to matriculation. However, traditional academic indicators alone may not identify those who ultimately assume leadership roles. These findings underscore the need for structured leadership development opportunities for all residents, regardless of early SLOE evaluations. Future research should explore whether intentional cultivation of leadership competencies throughout training can better support residents in achieving roles such as chief resident and beyond.

## INTRODUCTION

The emergency medicine (EM) Standardized Letter of Evaluation (SLOE) is a comprehensive assessment tool designed to provide meaningful comparative data to EM residency program directors. Given to EM-bound applicants during their fourth-year sub-internship, the SLOE incorporates both quantitative and qualitative data to evaluate an applicant’s clinical skills, non-cognitive characteristics, and overall competitiveness as an applicant.^1^ In addition to its role in selection, the SLOE also serves as a form of learner handover, bridging undergraduate and graduate medical education (UME/GME). In light of the call from the Coalition for Physician Accountability to strengthen the undergraduate to graduate medical education transition, the SLOE has the potential to support not only clinical handover but also the transfer of information about leadership-relevant domains.^2^

Historically, SLOEs have been considered one of the most important aspects of the applicant’s application when making decisions about interview invitations and placement on a program’s rank list.^3^–^7^ Despite the pivotal role SLOEs play in the selection process for EM residency programs, there is a notable gap in the literature regarding their ability to predict success in residency. Success can be a nebulous concept, varying significantly between program directors and institutions. Some may prioritize clinical acumen, while others may value leadership or teamwork abilities more highly.^8^ Research by Burkhardt et al highlights this variability, demonstrating that SLOEs have limited predictive power for residency success, often only relevant in specific scenarios.^9^

While the predictive power of SLOEs remains a topic of debate, little to no research has explored whether the qualities captured in these evaluations might also signal leadership potential that becomes relevant later in residency. Identifying such traits could help programs select residents who are not only clinically capable but also demonstrate the interpersonal and leadership skills valued in chief residents, such as communication, work ethic, fairness, and the ability to foster a collaborative environment.^10^,^11^ Many of the attributes assessed in the SLOE (initiative, teamwork, leadership, and professionalism) are foundational to residency success and may differentiate future leaders in EM.^12^ If these characteristics observed during fourth-year sub-internships correlate with chief resident selection, programs may gain a valuable framework for cultivating leadership potential earlier in training and making more informed decisions at the time of applicant selection.

Furthermore, understanding whether certain elements of the SLOE are associated with future residency leadership roles could have broad implications for residency recruitment and training. If certain traits assessed in medical students consistently translate into chief resident selection, this may prompt programs to emphasize these qualities more intentionally during both the application review process and resident development initiatives. By shifting the focus from short-term clinical competence to long-term leadership potential, programs may be able to better support residents’ professional growth, ensuring that those with leadership aspirations receive mentorship and opportunities to refine their skills. In this study we aim to examine the relationship between medical students’ SLOE evaluations and their likelihood of being selected as chief residents, thereby contributing to a more nuanced understanding of the SLOE’s role in forecasting not just immediate residency performance, but also future leadership in EM.

**Population Health Research Capsule**

What do we already know about this issue?

*Standardized Letters of Evaluation (SLOE) are key in emergency medicine (EM) residency selection, but their link to later leadership roles, such as chief resident, is unclear*.

What was the research question?

*Are early SLOE scores associated with later selection as chief resident?*

What was the major finding of the study?

*Future chief residents scored higher in teamwork (P = .002), overall comparison (P = .003), and rank list (P =.004)*.

How does this improve population health?

*Identifying early leadership traits can guide resident development, promoting stronger physician leaders and healthier healthcare systems*.

## METHODS

We performed a retrospective review of de-identified SLOEs from former and current EM residents at one large, urban, academic center residency program from 2015–2021. All identifiable elements of the SLOE, including applicant name, Association of American Medical Colleges) ID, and application year, were removed for data analysis. This study was reviewed and exempted by our institution’s institutional review board, and it was conducted and reported in accordance with the Strengthening the Reporting of Observational Studies in Epidemiology (STROBE) guidelines.

Each applicant was provided with a randomly generated study identifier, along with de-identified demographic elements (such as age during rotation, letter institution, sex, and whether they were chief or non-chief) linking all SLOEs collected to each respective resident. The SLOEs from residents who became chief residents were identified and labeled, although the names of those residents were removed from any further analysis of the data. We excluded non-EM letters of evaluation, such as off-service SLOEs, or elective rotation evaluations (ie, two-week ultrasound or emergency medical services electives).

The descriptive grading scales linked to each quantitative SLOE question were converted into numerical scales (Table 1) for data analysis. All data were extracted from previously completed SLOEs. Because the SLOE evaluates multiple distinct domains of performance, we analyzed individual items rather than using a composite score to allow exploration of which specific traits were most associated with eventual chief resident titles.

Each SLOE domain used in this analysis was originally assessed using a descriptive grading scale (eg, “Top 10%,” “Outstanding,” “Pass”). These were then converted to numerical scores for analysis using a predetermined conversion scheme (Table 1). We did not include “Fail” as part of the scale, as no student failed the rotation. The Cronbach alpha was not calculated as each scale was not part of a larger set of items measuring a single construct. However, sensitivity analyses helped validate our choice of numerical scale as our results remained consistent with different numerical conversions.

This retrospective review shares features with medical chart reviews, and we followed applicable best practices as outlined by Worster and Bledsoe.^13^ Specifically, data abstraction followed a standardized protocol, and numeric conversions were applied uniformly across all records. Reviewers were not blinded to the chief resident status, and inter-rater reliability was not formally assessed, which we acknowledge as potential sources of bias. Missing responses were excluded on a per-item basis and reported in the results. Additionally, we excluded any SLOEs lacking a descriptive grade or overall comparative rating (top 10%, upper-third, middle-third, or lower-third) from the analysis.

We analyzed data using IBM SPSS Statistics v29 (IBM Corporation, Armonk, NY). Although SLOE scores are ordinal, it is common practice to treat Likert-type scales as interval data when sample size is sufficient and distributions approximate normality.^14^,^15^ We confirmed robustness through sensitivity analyses, which yielded consistent results. A two-sided Student *t*-test for independent samples assuming unequal variance was used to determine mean differences in the quantitative questions for chief and non-chief residents. Additionally, we assessed sex differences between chiefs and non-chiefs using Pearson’s χ^2^ (2×2, Yates-corrected) and, to evaluate potential confounding, fit separate sex-adjusted logistic regressions for each Bonferroni-significant SLOE domain (chief status as the outcome), reporting odds ratios (OR) with 95% confidence intervals.

## RESULTS

We analyzed 243 SLOEs from 101 residents over a period of seven years. Overall, chief residents (n = 21; 20.8%) outperformed their non-chief counterparts (n = 80; 79.2%) in multiple domains, with several achieving statistically significant differences (Table 2).

Of the 10 quantitative SLOE questions converted to a numerical scale, three domains remained statistically significant after Bonferroni adjustment (*P* < .005). Chief residents received significantly higher ratings in teamwork ability (mean 2.712 vs 2.476, *P* = .002), overall ranking among other EM candidates recommended in that academic year (mean 2.904 vs 2.516, *P* = .003), and estimated final placement on the program’s rank list (mean 2.94 vs 2.541, *P* = .004). While chief residents had lower scores (mean 2.365 vs 2.136, *P* = .02) for the predicted amount of guidance they would require in residency, where a lower score was considered more favorable, and had higher ratings of predicted success given necessary guidance (mean 2.442 vs 2.241, *P* = .02). However, these domains did not meet the Bonferroni-corrected threshold following correction for multiple comparisons. Other assessed domains, including clinical reasoning, patient communication, and overall commitment to emergency medicine, did not show statistically significant differences between chief and non-chief residents.

Our program had a nearly even split of male and female chief residents over the study period (Table 3). Sex distribution did not differ between chiefs and non-chiefs (χ^2^ (1, N = 243) = 1.18, *P* = .28; Yates-corrected). After Bonferroni adjustment across 10 tests, the three SLOE domains remained significant: teamwork (*P* adj = .02), overall comparison to prior candidates (*P*_adj = .03), and estimated rank list placement (*P* adj = .04). In sex-adjusted simple logistic regressions using row-level scores, higher ratings in each domain were associated with increased odds of chief selection: teamwork OR 2.43 (95% CI, 1.27–4.64; *P* = .008), overall comparison OR 1.77 (1.19–2.62; *P* = 0.004), and rank list OR 1.83 (1.21–2.78; *P* = .004); the sex covariate was not statistically significant in these models.

Of note, while we report the number of independent vs group SLOEs and mean number of rotations for descriptive and transparency purposes, these variables were not included in our analyses.

## DISCUSSION

The SLOE remains one of the most influential components of an EM residency applicant’s portfolio, designed to assess clinical ability, professionalism, and leadership potential. While prior studies have questioned the SLOE’s ability to predict overall success in residency, in our study we sought to extend its value by examining whether SLOE-assessed traits correlate with future chief resident selection. Given the critical role chief residents play in residency programs as leaders, mentors, and administrative liaisons, identifying early indicators of leadership potential could provide valuable insights for both recruitment and resident development.

Our findings revealed that while many core qualities assessed in the SLOE did not show statistically significant differences between future chief and non-chief residents, certain key attributes stood out. Notably, chief residents scored significantly higher in teamwork-related domains, which is unsurprising given the collaborative nature of EM. The ability to effectively engage in team-based decision-making, communicate across disciplines, and balance multiple critical tasks is essential for both clinical success and leadership within an EM program. Given that teamwork is a fundamental component of high-functioning emergency departments, it is logical that those who excel in these areas may later emerge as natural leaders among their peers.

In contrast, traditional markers of individual success, such as work ethic, communication skills, and clinical competency, did not significantly differentiate future chiefs from non-chiefs. This finding suggests that chief residents are not necessarily the strongest performers in every domain from the outset of residency but may instead develop and refine leadership qualities over time. Rather than outperforming their peers in isolated areas, they may excel in self-directed learning, professional adaptability, and the ability to support and elevate those around them.

Importantly, these findings underscore the need to foster leadership development throughout residency, not just among those who ultimately become chief residents. While certain individuals may demonstrate leadership potential early in their training, residency programs should ensure that all residents have opportunities to cultivate and refine these skills. Structured leadership training, mentorship programs, and intentional opportunities for residents to take on leadership roles can provide valuable experience for future career growth, regardless of whether an individual is selected as a chief resident. By broadening leadership development efforts, programs can help all residents, regardless of their initial SLOE scores, enhance their ability to lead, collaborate, and contribute meaningfully to the field of EM.

By identifying early features of leadership potential, our findings highlight the importance of assessing and nurturing leadership qualities from the earliest stages of medical training. Emergency medicine residency programs may benefit from recognizing that leadership development extends beyond clinical acumen and should incorporate mentorship, structured leadership training, and opportunities for residents to cultivate skills in teamwork, adaptability, and self-directed learning. Future research should explore whether targeted interventions, such as early leadership curricula, mentorship initiatives, or self-assessment tools, could further support residents on the path to leadership roles within EM.

## LIMITATIONS

This study has several limitations. As a single-site study conducted in a three-year EM residency program, the findings may not be generalizable to programs with different structures or selection processes. Additionally, the study only captures associations between SLOE evaluations and chief resident selection but cannot determine causation. Many factors beyond early faculty assessments, such as mentorship, evolving leadership aspirations, and program-specific selection methods, likely influence chief resident selection. As previously noted in the “Methods” section, SLOE items are ordinal and were converted to numerical scores and analyzed as interval data to permit parametric tests; this assumes approximate equal spacing between categories and could influence effect size estimates.

Furthermore, our study did not assess the long-term leadership trajectory of chief residents beyond residency. It remains unknown whether these individuals pursued administrative roles or became national leaders in the field. Additionally, identifying chief resident predictors could introduce unintended bias if program directors begin selecting and grooming certain interns for leadership roles based on early performance rather than allowing leadership to develop organically. Future research should explore how structured leadership development programs can support all residents, regardless of early SLOE scores, in cultivating the skills necessary for future leadership roles.

Finally, the SLOE was revised in 2022 (eSLOE 2.0); this study used the legacy format. Of the domains that remained significant, teamwork and estimated rank-list placement are retained in the current letter, whereas overall comparison to prior candidates has no direct analogue, and the predicted guidance needs item is conceptually similar to the new anticipated guidance item. Therefore, generalization to eSLOE 2.0 should be cautious, although the core constructs remain applicable.

## CONCLUSION

The Standardized Letter of Evaluation offers valuable insights into the overall potential success of residency applicants, although their effectiveness in identifying specific leadership traits and future chief residents is limited. While there is no definitive predictor of who will assume the role of chief resident, our analysis indicates that applicants who demonstrate independence and strong teamwork skills are predicted to rank highly and exhibit overall success during residency and these skills are positively correlated with later selection as chief resident. Despite this association, we believe that programs should provide equal training and mentorship opportunities for all residents to develop and demonstrate these critical skills, so they can ensure that all residents, regardless of their initial SLOE scores, have the opportunity to grow into capable leaders.

## Figures and Tables

**Table 1:** Conversion of descriptive grading scales on Standardized Letter of Evaluation to numerical scores in a study examining associations between student evaluations and subsequent chief resident status in emergency medicine.

Questions	Descriptive scale	Numerical scale
1: Commitment to emergency medicine 2: Work ethic and willingness to assume responsibility 3: Ability to develop and justify an appropriate differential and treatment plan 5: Ability to work in a team 6: Ability to communicate a caring nature to patients 9: Overall comparison of applicant to previous years' applicants 10: Estimation of place on their rank list	Top 10%	4
	Top Third	3
	Middle Third	2
	Bottom Third	1
	
4: What grade did they receive?*	Honors	3
	High Pass	2
	Pass	1
7: How much guidance do you predict this applicant will need during residency?	Less than peers	3
	Same as peers	2
	More than peers	1
8: What is your prediction of success for this applicant?	Outstanding	3
	Excellent	2
	Good	1

**Table 2:** Comparison of quantitative Standardized Letter of Evaluation ratings between chief and non-chief residents in a study examining associations between student evaluations and subsequent chief resident status in emergency medicine.

Question	Chief status	Mean ± SD	N	P (unadjusted)	P (adjusted)
1. Commitment to emergency medicine; has carefully thought out this career choice.	Chief	2.558 ± 0.54	52	.78	1.00
	Non-chief	2.534 ± 0.54	191		
2. Work ethic, willingness to assume responsibility.	Chief	2.75 ± 0.44	52	.09	.90
	Non-chief	2.628 ± 0.53	191		
3. Ability to develop and justify an appropriate differential and cohesive treatment plan.	Chief	2.385 ± 0.57	52	.12	1.00
	Non-chief	2.246 ± 0.60	191		
4. What grade did they receive?	Chief	2.412 ± 0.57	51	.07	.70
	Non-chief	2.238 ± 0.68	189		
5. Ability to work with a team.	Chief	2.712 ± 0.46	52	.002	0.02
	Non-chief	2.476 ± 0.54	191		
6. Ability to communicate a caring nature to patients.	Chief	2.596 ± 0.50	52	.32	1.00
	Non-chief	2.518 ± 0.52	191		
7. How much guidance do you predict this applicant will need during residency?	Chief	2.365 ± 0.63	52	.02	0.20
	Non-chief	2.136 ± 0.62	191		
8. Given the necessary guidance, what is your prediction of success for the applicant?	Chief	2.442 ± 0.54	52	.02	0.20
	Non-chief	2.241 ± 0.55	191		
9. Compared to other EM residency candidates you have recommended in the last academic year; this candidate is in the:	Chief	2.904 ± 0.80	52	.003	0.03
	Non-chief	2.516 ± 0.81	190		
10. How highly would you estimate the candidate will reside on your rank list?	Chief	2.94 ± 0.87	50	.004	.04
	Non-chief	2.541 ± 0.79	183		

Multiple-comparison adjustment via Bonferroni across 10 tests (α = 0.05/10 = .005). Results meeting P < .005 prior to adjustment (P < 0.05 following adjustment) are considered statistically significant.

*SLOE*, Standard Letter of Evaluation; *EM*, emergency medicine.

**Table 3:** Demographic and Standardized Letter of Evaluation characteristics by chief resident status in a study examining associations between student evaluations and subsequent chief resident status in emergency medicine.

	Overall	Chief	Non-Chief
Men	66	12	54
Women	35	9	26
Mean Age	27.65	27.71	27.68
Independent SLOEs	63	14	49
Group SLOEs	180	38	142
Mean Number of Rotations	2.41	2.48	2.33

*SLOE*, Standardized Letter of Evaluation.
